# Effects of magnesium chloride on in vitro cholinesterase and ATPase poisoning by organophosphate (chlorpyrifos)

**DOI:** 10.1002/prp2.401

**Published:** 2018-04-30

**Authors:** Bamidele S. Ajilore, Adetayo A. Alli, Tolulope O. Oluwadairo

**Affiliations:** ^1^ Department of Biochemistry Faculty of Basic Medical Sciences College of Health Sciences Osun State University Osogbo Nigeria; ^2^ Department of Chemical Sciences Faculty of Basic and Applied Sciences College of Science, Engineering and Technology, Osun State University Osogbo Nigeria

**Keywords:** ATPases, chlorpyrifos, cholinesterase, Magnesium chloride, organophosphates

## Abstract

The present study investigated possible benefits of magnesium ion (as MgCl_2_) in organophosphorus poisoning targeting its ability to interact with substrates and membrane enzymes. Blood samples collected from volunteered healthy adult by venepuncture into anticoagulant test tubes containing EDTA were separated into plasma and red blood cell and divided into three groups namely: normal, pesticide only (0.25‐2.0 mmol/L chlorpyrifos) and pesticide (0.25‐2.0 mmol/L chlorpyrifos) + 0.1 mol/L MgCl_2_. Acetylcholinesterase, Na^+^/K^+^ ATPase and Ca^2+^ ATPase activities were evaluated. Results showed that Chlorpyrifos significantly (*P* < .05) reduced the levels of cholinesterase both in plasma and on red blood cells. Red blood cells Na^+^/K^+^ ATPase and Ca^2+^ ATPase were also significantly (*P* < .05) reduced by chlorpyrifos while MgCl_2_ counteracted effects of chlorpyrifos with significant (*P* < .05) increase in the levels of cholinesterase, Na^+^/K^+^ ATPase and Ca^2+^ ATPase. We concluded that MgCl_2_ neutralized effects of chlorpyrifos by promoting normal ATPase activities and inhibiting release of acetylcholine from cell.

**Abbreviation**
ATP: Adenosine triphosphate
Ca^2+^ ATPase: Calcium adenosine triphosphate
ChE: Cholinesterase
EDTA: Ethylenediaminetetraacetic acid
KCl: Potasium chloride
MgCl_2_: Magnesium chloride
MgSO_4_: Magnesium sulphate
NaCl: Sodium chloride
Na^+^/K^+^ ATPase: Sodium/potasiun adenosine triphosphate
TCA: Trichloroacetic acid
Tris‐HCl: Tris‐hydrochloride

## 1 INTRODUCTION

Pesticides are substances that are used to prevent, destroy, repel, and mitigate a host of unwanted organisms called pests.^1^ Pesticides are mostly not selective and their exposure are toxic to many nontargets including humans. The balance between their benefits and possible risks to their nontargets must be considered when they are being used. Organophosphorus pesticide poisoning has being a serious public health concern in developing countries.^2^ In northern part of Nigeria where farming is their major occupation, chlorpyriphos is traded under different brand names like Nuvan, perfect killer, Sniper, “Ota‐pia‐pia” (Hausa) and is being used indiscriminately as farm and household insecticides. Deaths recorded from accidental organophosphorus poisoning are less common than those from intentional poisoning, but are more common in areas where highly toxic organophosphorus pesticides are being used^3^, ^4^ to control pests on the farms, mostly in rural communities, and in the homes in urban areas. Every year, about one million unintentional and two million self poisonings with pesticides occur worldwide, and of these, approximately 200 000 die.^5^

Acute organophosphorus pesticide poisoning is a medical emergency.^6^ Atropine and oxime are the main antidotes of organophosphorous poisoning worldwide, but their efficacies have been an issue of debate.^5^ Although atropine can cause anticholinergic delirium in large doses, in a significantly organophosphorus‐poisoned patient, that toxicity is short‐lived as the pharmacologic duration of the atropine effect is far less than the cholinesterase inhibition from the organophosphate. Pralidoxime iodide in high doses can cause thyroid toxicity in patients. Organophosphorus pesticides inhibit both true cholinesterase (acetylcholinesterase) in synapses and on red cell membrane and pseudo(butyrilcholinesterase)cholinesterase in plasma by nucleophilic attack of the hydroxyl group of serine in the active sites of the enzymes . This results in phosphorylation and inactivation of the enzymes.^7^ Although the main toxic action of organophosphorus is as a result of inhibition of the active site of acetylcholinesterase, but some organophosphorus esters can cause a neuropathic anomaly which is not related to acute cholinergic effect^8^ but due to interaction of organophosphorus insecticides with activities of membrane ATPases. Nozdrenko et al.^9^ reported that chlorpyrifos inhibited Ca^2+,^Mg^2+^‐ATPase activity of sarcoplasmic reticulum of skeletal muscle.

Studies to find more effective antidotes for organophosphorus poisoning are in progress. The role of the magnesium cation in phosphoryl group transfer reactions has been reported.^10^ Inhibition of acetylcholinesterase by organophosphorus causes accumulation of acetylcholine at cholinergic synapses, overstimulation of cholinergic receptors and results in tremors and muscular twitching among other signs and symptoms of organophosphorus poisoning. Magnesium can relax muscle by blocking influx of calcium ions. It also interacts with substrates and enzymes by forming part of the active site using inner or outer sphere coordination. The acidity of Mg^2+^ aids hydrolysis and condensation reactions most especially phosphate ester hydrolysis and phosphoryl transfer. The aim of the present study was to investigate in vitro the possible benefits of magnesium ion (as MgCl_2_) in organophosphorus poisoning targeting its ability to interact with substrates and membrane enzymes.

## 2 MATERIALS AND METHODS

### 2.1 Materials

Adenosine triphosphate and acetylcholine iodide were obtained from Sigma Chemical Company. Chlorpyrifos was purchased from Nantong Jinling Agricultural Chemical Co., LTD. China. Sodium barbital, potassium dihydrogen phosphate, sodium chloride, Tris, EDTA, magnesium chloride, potassium chloride, calcium chloride, ammonium molybdate, aminonaphthol sulfonic acid (ANSA) were either obtained from BDH Ltd. Poole, England or Scharlab S.L. Spain. All reagents and chemicals used were of analytical grade.

### 2.2 Blood sample preparation

Blood samples were taken from volunteered healthy adult by venepuncture into anticoagulant test tubes containing EDTA and were used within 1 hour of collection. Plasma was obtained after centrifuging 4 mL of blood sample at 3000*g* for 10 minutes. Red blood cells were washed 3 times with 2 mL sodium phosphate buffer (0.1 mol/L, pH 8). The red blood cells were centrifuged between washes at 3000*g* for 10 minutes. Packed red blood cells were then diluted by hypotonic sodium phosphate buffer (6.7 mmol/L, pH 7.9) to facilitate hemolysis. This was followed by centrifugation at 3000*g* for 10 minutes. The supernatant was removed and pellet was resuspended in hypotonic phosphate buffer. The plasma was used to determine total cholinesterase activity while aliquots of red blood cell were used to determine total cholinesterase and ATPase activities.

### 2.3 Determination of cholinesterase activity

Plasma and erythrocyte cholinesterase activities were estimated using electrometric method described by Michel^11^ with modifications as shown in the protocol below:

**Table:** Protocol

	Normal control group	Pesticide only group	Pesticide + MgCl_2_ group
Samples (Plasma/red blood cell)	0.2 mL	0.2 mL	0.2 mL
dH_2_O	3 mL	3 mL	3 mL
Barbital PO_4_ buffer (pH 8.1)	3 mL	3 mL	3 mL
1N HCl (to adjust pH to 8.1)	Drops	Drops	Drops
Chlorpyrifos (0.25‐2.0 mmol/L)	—	0.1 mL	0.1 mL
0.1 mol/L MgCl_2_	—	—	0.1 mL

The pH of reaction mixtures was measured by pH meter (JENWAY 3520, Bibby Scientific Ltd., Essex, UK) as pH1. The reaction mixtures were incubated at 37°C for 20 minutes following addition of 0.1 mL 75 mmol/L acetylcholine iodide. The pH was measured again as pH2. All measurements were carried out in quintuplets. Cholinesterase activity was determined as follows:

$$\text{ChE activity}\left(\Delta \text{pH}\text{20 min}\right)=\left(\text{pH1}-\text{pH2}\right)-\text{pH blank}$$

Blank contained only water and barbital phosphate buffer. The percentage of cholinesterase enzyme inhibition was calculated as follows:

$$\begin{array}{cc} & \left(\text{ChE activity in normal control}\right)\\ & \;\;-\left(\text{ChE in pesticide}\;\;\text{magnesium chloride group}\right)\\ & \;\;÷\left(\text{ChE activity in normal control}\right)\times 100 \end{array}$$

The 50% inhibitory concentration (IC_50_) was also calculated.

### 2.4 Determination of ATPase activity

The activity of Na^+^/K^+^‐dependent ATPase was determined by the method of Bonting,.^12^ In this assay, 0.1 mL of different concentrations (0.25‐2.0 mmol/L) chlorpyrifos was added to 0.1 mL aliquot of red cell in test tube containing 1 mL of 184 mmol/L Tris‐HCl buffer (pH 7.5), 0.2 mL of 50 mmol/L MgSO_4_, 0.2 mL of 50 mmol/L KCL, 2 mL of 600 mmol/L NaCl, 0.2 mL of 1 mmol/L EDTA, and 0.2 mL of 10 mmol/L ATP and incubated for 15 minutes at 37°C. The reaction was stopped by the addition of 9 mL of ice‐cold 5% TCA. The amount of Pi liberated was estimated in protein‐free supernatant according to the method of Fiske and Subbarow^13^ at 412 nm.

The activity of Ca^2+^‐ATPase was assayed according to the method of Hjertan and Pan.^14^ Briefly, 0.1 mL of different concentrations (0.25‐2.0 mmol/L) chlorpyrifos was added to 0.1 mL aliquot of red cell in a test tube containing 0.1 mL of 125 mmol/L Tris‐HCl buffer (pH 8), 0.1 mL of 50 mmol/L CaCl_2_, and 0.1 mL of 10 mmol/L ATP. The contents were incubated at 37°C for 15 minute. The reaction was arrested by the addition of 9 mL of ice‐cold 5% TCA and centrifuged. The amount of Pi liberated was estimated in supernatant at 412 nm.

### 2.5 Statistical analysis

Data obtained were analyzed using One‐Way Analysis of Variance (SPSS version 20.0). Levene statistic was used for tests of homogeneity of variance. Duncan was used for multiple comparisons and homogenous subsets. Results were considered to be statistically significant when *P* < .05.

## 3 RESULTS

**Figure 1:**
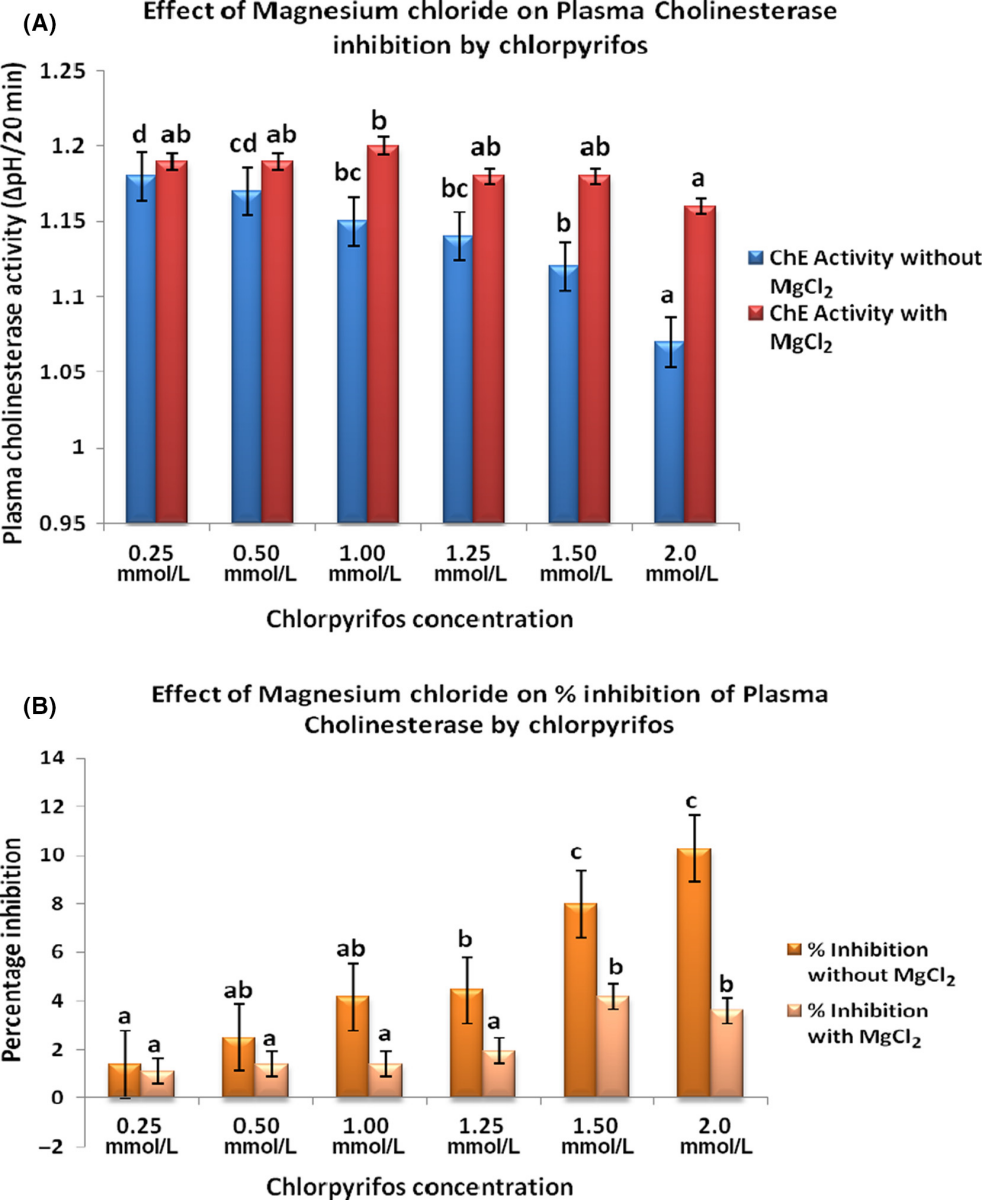
A and B showed activities of acetylcholinesterase and corresponding percentage inhibitions in plasma respectively. Values are expressed as mean ± SD (n = 5). Duncan superscripts a, ab, b, bc, c, cd, d are significance homogenous subsets of means within groups. Bars of the same legend with different Duncan superscripts are statistically significant at *P* < .05

**Figure 2:**
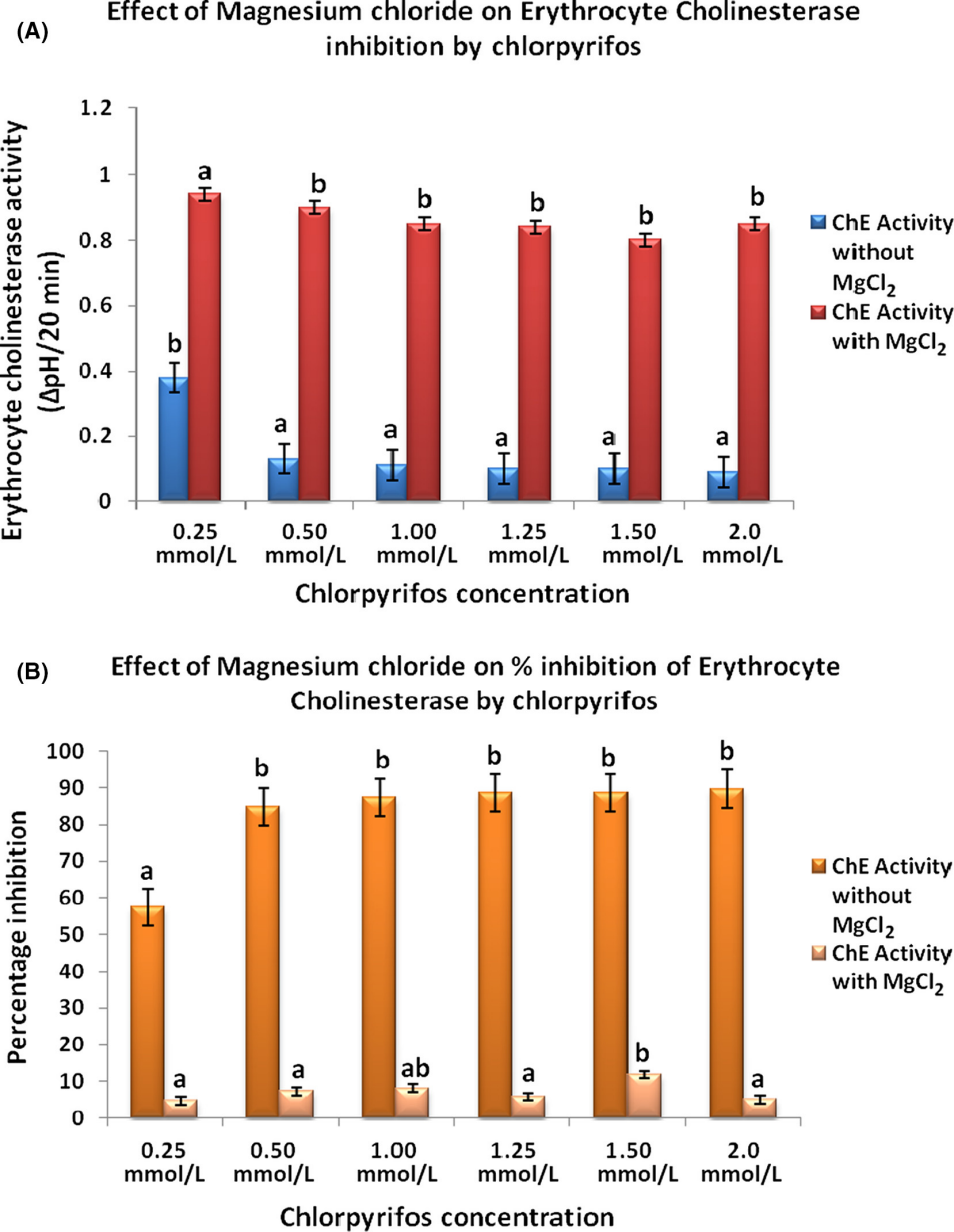
A and B showed activities of acetylcholinesterase and corresponding percentage inhibitions on red blood cell respectively. Values are expressed as mean ± SD (n = 5). Duncan superscripts a, ab, b, bc, c, cd, d are significance homogenous subsets of means within groups. Bars of the same legend with different Duncan superscripts are statistically significant at *P* < .05

**Figure 3:**
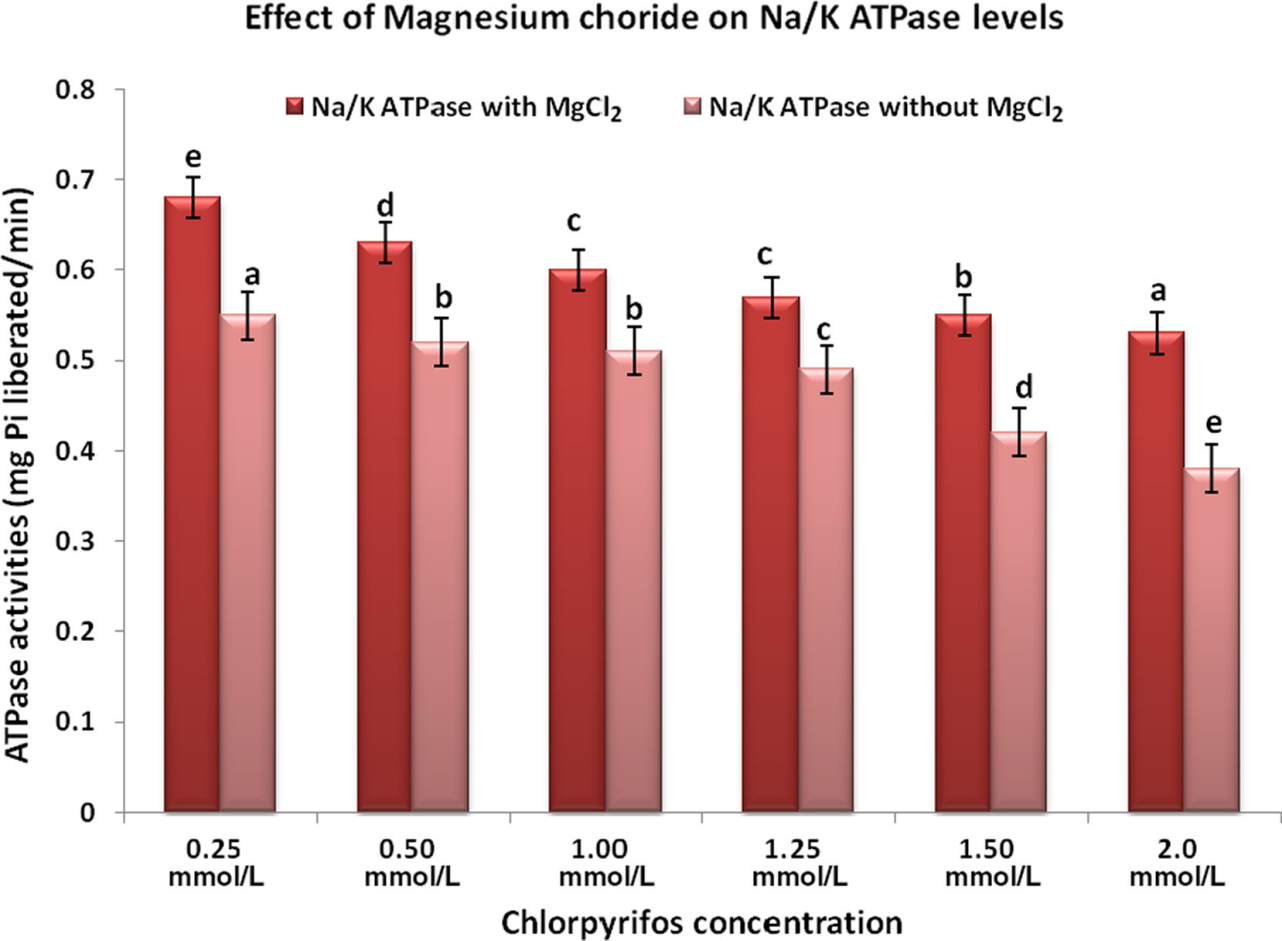
Na^+^/K^+^ ATPase activities in the presence and absence of MgCl_2_. Values are expressed as mean ± SD (n = 5). Duncan superscripts a, ab, b, bc, c, cd, d are significance homogenous subsets of means within groups. Bars of the same legend with different Duncan superscripts are statistically significant at *P* < .05

**Figure 4:**
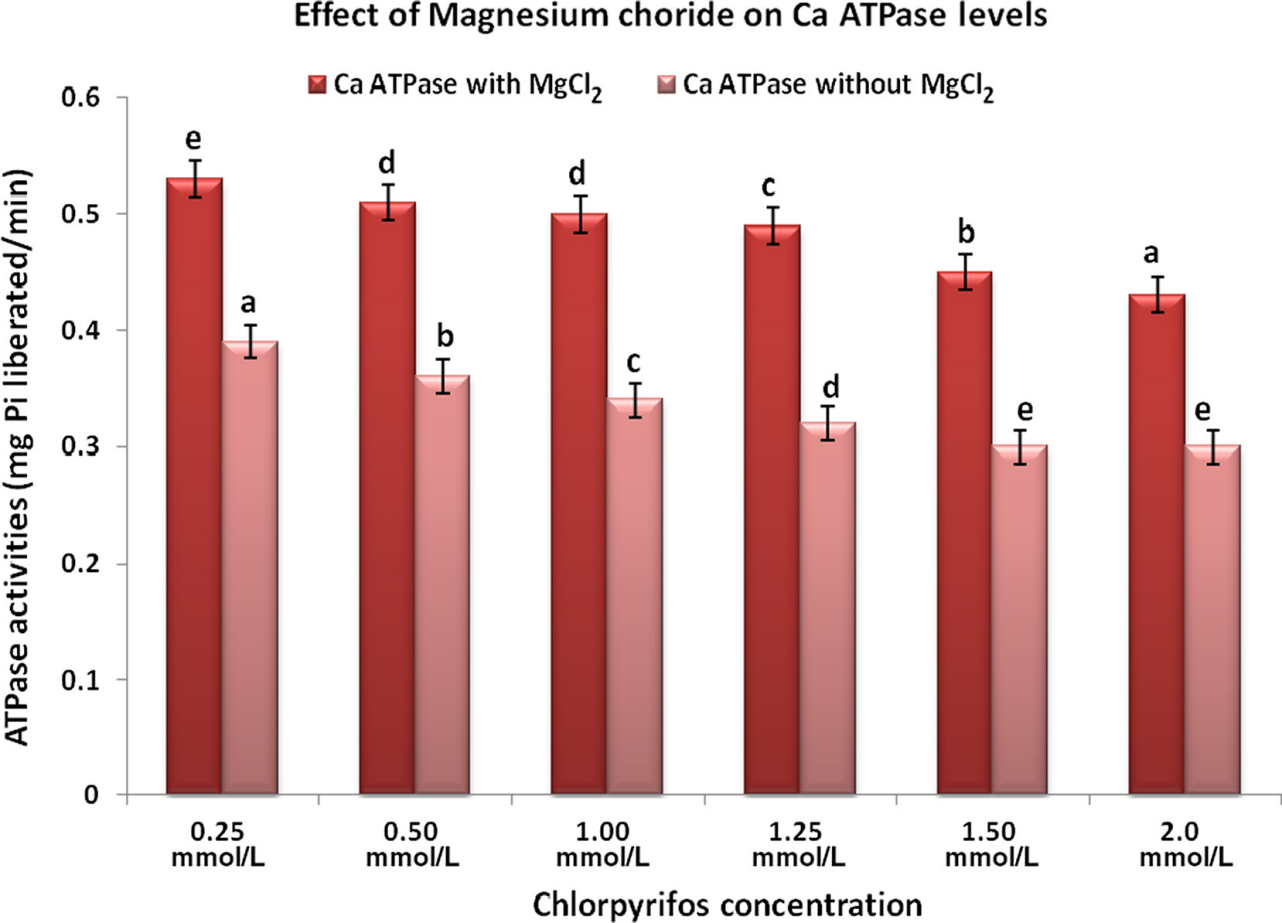
Ca^2+^ ATPase activity in the presence and absence of MgCl_2_. Values are expressed as mean ± SD (n = 5). Duncan superscripts a, ab, b, bc, c, cd, d are significance homogenous subsets of means within groups. Bars of the same legend with different Duncan superscripts are statistically significant at *P* < .05

To determine whether magnesium chloride has beneficial effect in organophosphate‐induced toxicity or not, we performed this study with a fixed dose of MgCl_2_ (0.1 mol/L) at increasing concentration of chlorpyrifos (0.25‐2.0 mol/L). The concept was to assess pharmacological effectiveness of fixed dose of MgCl_2_ beyond 50% inhibition of the enzyme by chlorpyrifos. Figure 1A and B showed activities of acetylcholinesterase and corresponding percentage inhibitions in plasma respectively. Figure 2A and B showed activities of acetylcholinesterase and corresponding percentage inhibitions in red blood cell respectively. Figures 3 and 4 showed activities of Na^+^/K^+^‐ATPase and Ca^2+^‐ATPase respectively.

### 3.1 Levels of acetylcholinesterase activities

Chlorpyrifos significantly (*P* < .05) reduced both plasma and red blood cell cholinesterase in a dose‐dependent manner. The percentage inhibition of cholinesterase in the absence of MgCl_2_ was least at 0.25 mmol/L and highest at 2.0 mmol/L concentrations of the pesticide. Irrespective of increasing chorpyrifos concentration, addition of a fixed dose 0.1 mol/L MgCl_2_ significantly kept cholinesterase activities at near‐constant high levels and the percentage inhibition at near‐constant low levels. The 50% inhibitory concentrations (IC_50_) of chlorpyrifos in plasma and red blood cell were 1.35 mmol/L and 0.20 mmol/L respectively.

### 3.2 Levels of ATPase activities

Figures 3 and 4 showed Na^+^/K^+^ ATPase and Ca^2+^ ATPase activities in the presence and absence of MgCl_2_. At a fixed dose 0.1 mol/L, MgCl_2_ significantly (*P* < .05) kept levels of Na^+^/K^+^ ATPase and Ca^2+^ ATPase higher irrespective of increase in pesticide concentrations while chlorpyrifos significantly (*P* < .05) reduced activities of the two membrane enzymes.

## 4 DISCUSSION AND CONCLUSIONS

Chlorpyrifos is an organophosphate pesticide widely used to control insect pests on the farms and in the house. Self‐poisoning from chorpyrifos use is an important clinical problem in developing world.^6^ The present study investigated benefits of magnesium ion (as MgCl_2_) in organophosphorus poisoning targeting its ability to interact with substrates and membrane enzymes. The method used in this study to measure blood acetylcholinesterase activity is based on hydrolysis of acetycholine.^11^, ^15^ Our study showed that chlorpyrifos significantly (*P* < .05) reduced the levels of acetylcholinesterase in plasma and on red cell membrane in a dose‐dependent manner. Organophosphorus insecticides inhibit acetylcholinesterase by nucleophilic attack on the active site of the enzyme which results in phosphorylation of the serine‐OH group in the active site and subsequent inhibition of the enzyme.^7^ The formation of OP‐AChE complex formed if not reversed quickly leads to a dealkylation process that causes reduction in the levels of acetylcholinesterase as observed in our study. At this point, atropine and oximes which are common antidotes being used in managing organophosphate insecticide poisoning could not be effective.^7^, ^16^ Magnesium chloride produces divalent cation in solution which lowers pKa of nucleophiles,^17^ and may result in lower charge repulsion between phosphoryl moiety of organophospate and serine‐OH group of AChE, and then reactivation of the enzyme. The subsequent magnesium ion acidity might have resulted in increased hydrolysis of acetycholine and significant (*P* < .05) reductions in percentage inhibition of plasma and red blood cell cholinestearase observed in this study.

ATPases are groups of membrane‐bound enzymes involved in the breaking down of ATP to ADP and free Pi. The dephosphorylation is coupled with release of energy needed by the enzyme to drive endergonic reactions. Inhibition of red blood cell membrane Na^+^/K^+^ ATPase in this study was dose‐dependent. Increase in the concentration of chlorpyrifos significantly (*P* < .05) reduced the activity of the enzyme. Inhibition of Na^+^/K^+^ ATPase by chlorpyrifos may be due to its interaction with the process of dephosphorylation of the enzyme. ATPase is a specific feature of the interior part of Na^+^‐ K^+^ pump responsible for establishing a negative electrical voltage inside the cells that maintains resting membrane potential.^18^ Increasing concentration of chlorpyrifos in this study significantly (*P* < .05) reduced activity of Ca^2+^ ATPase. The plasma membrane calcium pump requires magnesium for normal export of calcium and keeps intracellular calcium level low.^19^ Inhibition of this enzyme by chlorpyrifos causes elevated intracellular calcium,^20^ increased exocytosis of acetycholine, and is associated with signs and symptoms of neuropathy seen in organophosphate toxicity.^21^ Magnesium chloride which is widely used inorganic salt in chemistry and molecular biology as a source of magnesium ion^22^ is an important cofactor in many enzymes, including Ca^2+^ ATPase. Magnesium chloride in this study significantly (*P* < .05) increased the activity of Ca^2+^ ATPase, which might have led to decrease in intracellular calcium and indirectly inhibited acetylcholine release from the cell.

The results of our study concluded that chlorpyrifos toxicity inhibited acetylcholinesterase, Na^+^/K^+^ ATPase and Ca^2+^ ATPase activities. MgCl_2_ on the other hand neutralized effects of chlorpyrifos poisoning by promoting normal ATPase activities and inhibiting release of acetylcholine from cell.

## DISCLOSURE

Authors declare no conflict of interest.
